# Linking prison health care data to other health data: A novel data linkage study in Northern Ireland

**DOI:** 10.23889/ijpds.v8i2.2341

**Published:** 2023-09-14

**Authors:** Janine Cooper, Dermot O’Reilly, Trish Kelly, Richard Kirk, Ruth Gray, Michael Donnelly

**Affiliations:** 1 Queen's University Belfast, Belfast, United Kingdom; 2 Administrative Data Research Centre Northern Ireland (ADRC NI), Belfast, United Kingdom; 3 Healthcare in Prison, South Eastern Health and Social Care Trust, Belfast, United Kingdom

## Objectives

We describe the progress made in the development of a novel linked database designed to improve understanding about the health, mental health, health service use and mortality risk of people following their release from prison in Northern Ireland (NI).

## Methods

This is a collaborative project between the ADRC-NI and the NI Healthcare in Prisons service (HIPS) - South Eastern Health and Social Care Trust (SEHSCT). Data about the health of all adult prisoners between 2012 and 2021 (HIPS) will be linked to a healthcare population spine (National Health Application and Infrastructure Services - NHAIS) via a Health and Care Number (HCN), a unique health identifier for each patient in the NHS in Northern Ireland. Data will subsequently be linked to prescribing data (Enhanced Prescribing Database - EPD), in-patient services data (mental health) and mortality data (General Register Office - GRO).

## Results

Ethical and governance approvals have been obtained. A stepwise approach to the development of this novel health database will be presented. Data cleaning has been undertaken by the HIPS team and records with missing HCNs have been identified and updated. Extraction of prison health data is underway for N=14,898 individuals and N=34,213 custodial episodes. Individuals may have more than one prison episode during the study period. Electronic and manual modes were used to extract 25 health-related variables from prison records. Data will be transferred to the Honest Broker Service (HBS), the trusted research environment for health and social care in NI, for data de-identification and linkage, and data access for analysis. Preliminary results and lessons for other related data-linkage projects will be presented and discussed.

## Conclusion

We will describe our progress regarding the development of a novel health dataset comprising routinely collected administrative data and our work about prisoner health. We will report about our experience of assessing data access, cleaning, extracting, and analysing data and the linkage possibilities with respect to the prison health dataset.

